# Impact of glycated hemoglobin on 2-year clinical outcomes in elderly patients with atrial fibrillation: sub-analysis of ANAFIE Registry, a large observational study

**DOI:** 10.1186/s12933-023-01915-3

**Published:** 2023-07-12

**Authors:** Yasuo Terauchi, Hiroshi Inoue, Takeshi Yamashita, Masaharu Akao, Hirotsugu Atarashi, Takanori Ikeda, Yukihiro Koretsune, Ken Okumura, Shinya Suzuki, Hiroyuki Tsutsui, Kazunori Toyoda, Atsushi Hirayama, Masahiro Yasaka, Takenori Yamaguchi, Satoshi Teramukai, Tetsuya Kimura, Yoshiyuki Morishima, Atsushi Takita, Wataru Shimizu

**Affiliations:** 1 Department of Endocrinology and Metabolism, Graduate School of Medicine, Yokohama City University, Kanagawa, Japan; 2 Saiseikai Toyama Hospital, Toyama, Japan; 3 The Cardiovascular Institute, Tokyo, Japan; 4 Department of Cardiology, National Hospital Organization Kyoto Medical Center, Kyoto, Japan; 5 AOI Hachioji Hospital, Tokyo, Japan; 6 Department of Cardiovascular Medicine, Toho University Faculty of Medicine, Tokyo, Japan; 7 National Hospital Organization Osaka National Hospital, Osaka, Japan; 8 Division of Cardiology, Saiseikai Kumamoto Hospital Cardiovascular Center, Kumamoto, Japan; 9 Department of Cardiovascular Medicine, Faculty of Medical Sciences, Kyushu University, Fukuoka, Japan; 10 Department of Cerebrovascular Medicine, National Cerebral and Cardiovascular Center, Osaka, Japan; 11 Osaka Police Hospital, Osaka, Japan; 12 Department of Cerebrovascular Medicine and Neurology, National Hospital Organization Kyushu Medical Center, Fukuoka, Japan; 13 Department of Biostatistics, Graduate School of Medical Science, Kyoto Prefectural University of Medicine, Kyoto, Japan; 14 Primary Medical Science Department, Daiichi Sankyo Co., Ltd, Tokyo, Japan; 15 Data Intelligence Department, Daiichi Sankyo Co., Ltd, Tokyo, Japan; 16 Department of Cardiovascular Medicine, Graduate School of Medicine, Nippon Medical School, Tokyo, Japan

**Keywords:** Atrial fibrillation, Diabetes mellitus, Elderly patients, Stroke, Systemic embolism, Oral anticoagulants, Direct oral anticoagulants, Observational study, Major bleeding, Intracranial hemorrhage

## Abstract

**Background:**

This ANAFIE Registry sub-analysis investigated 2-year outcomes and oral anticoagulant (OAC) use stratified by glycated hemoglobin (HbA1c) levels among Japanese patients aged ≥ 75 years with non-valvular atrial fibrillation (NVAF) with and without clinical diagnosis of diabetes mellitus (DM).

**Methods:**

The ANAFIE Registry was a large-scale multicenter, observational study conducted in Japan; this sub-analysis included patients with baseline HbA1c data at baseline. The main endpoints evaluated (stroke/systemic embolic events [SEE], major bleeding, intracranial hemorrhage, cardiovascular death, all-cause death, and net clinical outcome [a composite of stroke/SEE, major bleeding, and all-cause death]) were stratified by HbA1c levels (< 6.0%; 6.0% to < 7.0%; 7.0% to < 8.0%; and ≥ 8.0%).

**Results:**

Of 17,526 patients with baseline HbA1c values, 8725 (49.8%) patients had HbA1c < 6.0%, 6700 (38.2%) had 6.0% to < 7.0%, 1548 (8.8%) had 7.0% to < 8.0%, and 553 (3.2%) had ≥ 8.0%. Compared with other subgroups, patients with HbA1c ≥ 8.0% were more likely to have lower renal function, higher CHA_2_DS_2_-VASc and HAS-BLED scores, higher prevalence of non-paroxysmal AF, and lower direct OAC (DOAC) administration, but higher warfarin administration. The HbA1c ≥ 8.0% subgroup had higher event rates for all-cause death (log-rank *P* = 0.003) and net clinical outcome (log-rank *P* = 0.007). Similar trends were observed for stroke/SEE. In multivariate analysis, risk of all-cause death (adjusted hazard ratio [aHR]: 1.46 [95% confidence interval 1.11–1.93]) and net clinical outcome (aHR 1.33 [1.05–1.68]) were significantly higher in the HbA1c ≥ 8.0% subgroup. No significant differences were observed in risks of major bleeding or other outcomes in this and other subgroups. No interaction was observed between HbA1c and OACs. Use/non-use of antidiabetic drugs was not associated with risk reduction; event risks did not differ with/without injectable antidiabetic drugs.

**Conclusions:**

Among elderly Japanese patients with NVAF, only HbA1c ≥ 8.0% was associated with increased all-cause death and net clinical outcome risks; risks of the events did not increase in other HbA1c subgroups. Relative event risks between patients treated with DOACs and warfarin were not modified by HbA1c level.

**Trial registration:**

UMIN000024006; date of registration: September 12, 2016.

**Supplementary Information:**

The online version contains supplementary material available at 10.1186/s12933-023-01915-3.

## Background

Aging is a known risk factor for atrial fibrillation (AF), and the prevalence of AF increases with age, with more marked increases in people aged over 60 years [1–3]. The prevalence of diabetes mellitus (DM) also increases with age, particularly over the age of 65 years [4]. DM is a risk factor for developing stroke and systemic embolic events (SEE) in patients with AF [5], and it is among the factors used for clinical risk stratification for predicting stroke and thromboembolism in AF, namely the CHADS_2_ or CHA_2_DS_2_-VASc scores [6]. Among patients with AF, high proportions present with DM as a comorbidity; such patients tend to have worse outcomes and increased mortality compared with patients who do not have DM [7].

Glycated hemoglobin (HbA1c), an indicator of the average blood glucose level over the previous 2 to 3 months, is an indicator of glycemic control. It has been reported that glycemic control to HbA1c < 7.0% is important for suppressing the development and progression of microvascular complications [8, 9]. HbA1c targets for non-elderly patients with DM are HbA1c < 6.0% for patients thought to achieve glycemic control through diet and exercise therapy alone or those likely to achieve it while undergoing pharmacologic therapy without adverse reactions; HbA1c < 7.0% to prevent complications; HbA1c < 8.0% for those in whom intensive therapy is difficult because of the risk of hypoglycemia, among other reasons. For elderly patients with diabetes, however, the Japan Diabetes Society and Japan Geriatrics Society Joint Committee recommended in 2017 that patients be classified into three categories depending on the patient’s background characteristics and health status, such as age, cognitive function, and basic/instrumental activities of daily living, and that patients in each category be further divided into those receiving and those not receiving drugs potentially associated with severe hypoglycemia, such as insulin, sulfonylureas, and glinides [10]. Furthermore, the guideline specified the upper and lower limits of HbA1c to prevent diabetic complications and severe hypoglycemia. According to this guideline, the HbA1c target is < 8.5% for patients at risk of developing adverse reactions to multi-drug combination therapy or in those with serious comorbidities or poor social support [11].

Several studies have evaluated the incidence rates of adverse clinical outcomes in AF patients with and without DM, and the effects of oral anticoagulant (OAC) therapy on the incidence rates of outcomes [12–14]. The ENGAGE AF-TIMI 48 trial reported that, compared with patients with AF and without DM, patients with DM had a similar rate of stroke/SEE (adjusted hazard ratio [aHR] 1.08; *P* = 0.28) but a significantly higher risk of major bleeding (aHR 1.28; *P* < 0.001) after multivariate adjustments [12]. In the RELY trial, AF patients with DM had a stroke or SEE more frequently than patients without DM [13]. In the ROCKET-AF trial, an adjusted analysis suggested that AF patients with DM had 1.3-, 1.5-, and 1.9-fold higher 2-year rates of stroke, vascular mortality, and myocardial infarction, respectively, than AF patients without DM [14]. However, most AF patients in these studies were aged < 75 years and tended to be compared by the presence or absence of DM. A recent study investigated the association between HbA1c levels and the risk of stroke/SEE and major bleeding among patients with AF treated with or without OACs as well as the effectiveness and safety of warfarin vs. DOACs in a subgroup analysis stratified by different HbA1c levels [15]. The risk of events increased significantly with HbA1c levels above 6.5%. That study also found that, compared with warfarin, DOACs were more effective and safer in all HbA1c levels. The study indicated that glycemic control to achieve a more stringent target of HbA1c < 6.5% may be warranted in AF patients. However, it is important to note that patients in that study had a mean age between 68.9 and 71.2 years [15] and were younger than the target population of ANAFIE [14]. Thus, few studies have evaluated the incidence of main clinical outcome by HbA1c level and efficacy and safety by OAC status in elderly AF patients, particularly in those ≥ 75 years of age.

The All Nippon AF in the Elderly (ANAFIE) Registry is an observational prospective study conducted at multiple centers in Japan focusing on elderly patients (aged ≥ 75 years) with non-valvular AF (NVAF) to collect real-world data on their clinical status, anticoagulant therapy status, and prognosis with or without anticoagulation [14]. The baseline characteristics [16] and main outcomes at the 2-year follow-up have been reported [17]. The objective of this sub-analysis of the ANAFIE Registry was to investigate the association between HbA1c levels and outcomes among elderly Japanese patients with NVAF and to compare the risks of outcomes in patients treated with DOAC vs. warfarin for each HbA1c level.

## Methods

### Study design

The ANAFIE Registry was a large-scale multicenter, observational study conducted between October 2016 and January 2020 in Japan with the participation of 1273 medical facilities that enrolled elderly patients (≥ 75 years old) diagnosed with NVAF. The rationale and details of the study design, including disease classifications, diagnostic criteria, and assessments based on the AF guidelines of the Japanese Circulation Society and Japanese Heart Rhythm Society have been published previously [14, 18]. Data collection took place at baseline. Collected data included patient demographics, medical history including information on comorbidities (e.g., severe hepatic disease, hyperuricemia, heart failure and/or reduced left ventricular ejection fraction), occurrence of AF (i.e., type of AF, date and method of diagnosis, symptoms, and treatment decisions); history of catheter ablation, type of anticoagulant used; use of drugs other than anticoagulants (e.g., use of antiarrhythmic drugs, anti-platelet agents, proton pump inhibitors, and P-glycoprotein inhibitors); polypharmacy; and blood coagulation test under warfarin [14]. The patient follow-up was planned for a 2-year duration. As this was not an interventional study, the decision to prescribe OACs (e.g., warfarin or direct OACs [DOACs]) or not (no OACs), as well as oral or injectable antidiabetic drugs, depended entirely on the treating physician’s judgement.

### Patients

The overall Registry included adult men and women diagnosed with NVAF based on electrocardiogram results who could attend hospital visits. This sub-analysis included patients with available data on HbA1c measurements at baseline. National Glycohemoglobin Standardization Program HbA1c values were used. Clinical diagnosis of DM by treating physicians was not an inclusion criterion for this sub-analysis.

The main exclusion criteria for the overall Registry were patients with NVAF who were participating in other studies; had undergone artificial valve replacement; had a recent history of a cardiovascular event such as stroke, myocardial infarction, cardiac intervention, heart failure, any bleeding leading to hospitalization within 1 month prior to enrollment, and life expectancy < 1 year; and deemed inappropriate for study participation by the investigator.

### Study endpoints

The main endpoints evaluated were stroke/SEE, major bleeding, intracranial hemorrhage, cardiovascular death, all-cause death, and net clinical outcome (a composite endpoint of stroke/SEE, major bleeding, and all-cause death). Endpoints were stratified by HbA1c levels (HbA1c < 6.0%, 6.0% to < 7.0%, 7.0% to < 8.0%, and ≥ 8.0%). Subgroup analyses were also performed by type of OAC (warfarin and DOACs) for all patients and by type of antidiabetic drug (oral and injectable antidiabetic agents) for patients with a clinical diagnosis of DM.

### Statistical analysis

Categorical variables were analyzed using frequency tables. Summary statistics were calculated for continuous variables and included n, mean, and standard deviation. Analysis of variance was used for comparison of continuous variables, and the chi-squared test, for categorical variables.

The probability of event occurrence during the 2-year follow-up period for each HbA1c level was estimated using the Kaplan–Meier method, and the corresponding *P*-values were calculated using the log-rank test. Incidence rates per 100 person-years with 95% confidence intervals (CIs) were also estimated. Hazard ratios (HR) were estimated using the Cox proportional hazards model adjusted by prognostic factors among the four HbA1c groups. The variables selected for this analysis were those possibly associated with the selection of the anticoagulant therapy or the incidence of outcomes [17]. A *P*-value < 0.05 was considered statistically significant. The statistical software used for analysis was SAS version 9.4 (SAS Institute, Tokyo, Japan).

## Results

### Patient disposition and characteristics

Of the 32,275 patients enrolled in the ANAFIE Registry, 17,526 patients with known HbA1c values at baseline were included in this analysis. The median (Q1–Q3) follow-up duration of this study was 2.0 (1.92–2.00) years. By HbA1c group at baseline, 8725 (49.8%) patients had HbA1c < 6.0%, 6700 (38.2%) had 6.0% to < 7.0%, 1548 (8.8%) had 7.0% to < 8.0%, and 553 (3.2%) had ≥ 8.0%.

In the overall ANAFIE population [16, 17], patients had a mean age of 81.5 years; 57.3% were male; mean CHA_2_DS_2_-VASc score, 4.5; mean HAS-BLED score,1.9; proportion of patients with paroxysmal AF, 42.1%; mean creatinine clearance (CrCl), 48.4 mL/min; and OAC use, 92.4% (DOACs, 66.9%; warfarin, 25.5%). Patients with HbA1c data at baseline (n = 17,526) had similar clinical characteristics, and 40.4% had a clinical diagnosis of diabetes mellitus (Table 1). Regarding the use of antidiabetic drugs, 24.6% were taking oral antidiabetic drugs, and 3.6% were taking injectable antidiabetic drugs (insulin [3.2%] and glucagon-like peptide-1 [GLP-1] receptor agonists [0.4%]). The most common oral antidiabetic drugs were dipeptidyl peptidase-4 (DPP-4) inhibitors (19.2%), sulfonylureas (6.1%), alpha-glucosidase inhibitors (4.9%), sodium glucose co-transporter 2 (SGLT2) inhibitors (2.1%), thiazolidinediones (1.9%), and others (5.0%) (Table 1).

**Table 1:** Characteristics of 17,526 patients at baseline by HbA1c levels

	Total	HbA1c, %				*P*-value*
		< 6.0 (n = 8725)	6.0 to < 7.0 (n = 6700)	7.0 to < 8.0 (n = 1548)	≥ 8.0 (n = 553)	
Male	10,351 (59.1)	4955 (56.8)	4111 (61.4)	981 (63.4)	304 (55.0)	< 0.001
Age (years)	81.3 ± 4.7	81.4 ± 4.8	81.1 ± 4.6	80.8 ± 4.4	81.7 ± 4.6	< 0.001
≥ 85 years	4336 (24.7)	2288 (26.2)	1566 (23.4)	332 (21.4)	150 (27.1)	-
Body mass index (kg/m^2^)	23.6 ± 3.6	23.2 ± 3.5	24.0 ± 3.7	24.4 ± 3.6	24.3 ± 4.0	< 0.001
Systolic blood pressure (mmHg)	127.6 ± 16.8	127.6 ± 16.7	127.3 ± 16.8	128.2 ± 16.7	128.0 ± 17.8	0.297
Creatinine clearance (mL/min)	49.6 ± 18.7	49.2 ± 17.4	50.3 ± 20.5	50.2 ± 17.2	47.6 ± 18.6	< 0.001
< 50 mL/min	8527 (48.7)	4343 (49.8)	3137 (46.8)	745 (48.1)	302 (54.6)	-
Hemoglobin (g/dL)	13.0 ± 1.7	12.9 ± 1.7	13.0 ± 1.7	13.1 ± 1.8	13.1 ± 1.9	< 0.001
CHA_2_DS_2_-VASc score	4.6 ± 1.4	4.3 ± 1.4	4.8 ± 1.4	5.3 ± 1.3	5.5 ± 1.4	< 0.001
HAS-BLED score	1.9 ± 0.9	1.9 ± 0.9	1.9 ± 0.9	2.1 ± 0.9	2.1 ± 0.9	< 0.001
History of major bleeding	802 (4.6)	394 (4.5)	315 (4.7)	65 (4.2)	28 (5.1)	0.773
AF type						
Paroxysmal	7097 (40.5)	3802 (43.6)	2538 (37.9)	567 (36.6)	190 (34.4)	< 0.001
Persistent/Long-standing persistent	5341 (30.5)	2583 (29.6)	2062 (30.8)	520 (33.6)	176 (31.8)	-
Permanent	5088 (29.0)	2340 (26.8)	2100 (31.3)	461 (29.8)	187 (33.8)	-
Anticoagulant	16,264 (92.8)	8000 (91.7)	6287 (93.8)	1458 (94.2)	519 (93.9)	< 0.001
DOAC	11,921 (68.0)	6078 (69.7)	4450 (66.4)	1048 (67.7)	345 (62.4)	< 0.001
Warfarin	4336 (24.7)	1918 (22.0)	1836 (27.4)	410 (26.5)	172 (31.1)	< 0.001
TTR	75.8 ± 29.5	75.0 ± 30.1	76.8 ± 28.8	75.6 ± 30.3	74.5 ± 29.4	0.342
**Antidiabetic medication**						
Oral diabetes medication	4318 (24.6)	468 (5.6)	2277 (35.1)	1149 (75.1)	424 (77.4)	< 0.001
Sulfonylurea	1073 (6.1)	72 (0.9)	424 (6.5)	386 (25.2)	191 (34.9)	< 0.001
Alpha-glucosidase inhibitor	851 (4.9)	87 (1.0)	421 (6.5)	250 (16.3)	93 (17.0)	< 0.001
Thiazolidinedione	333 (1.9)	36 (9.4)	165 (2.5)	90 (5.9)	42 (7.7)	< 0.001
Dipeptidyl peptidase-4 inhibitor	3360 (19.2)	343 (4.1)	1773 (27.3)	911 (59.5)	333 (60.8)	< 0.001
Sodium glucose co-transporter 2 inhibitor	364 (2.1)	21 (0.3)	170 (2.6)	115 (7.5)	58 (10.6)	< 0.001
Others	874 (5.0)	50 (0.6)	443 (6.8)	286 (18.7)	95 (17.3)	< 0.001
Insulin	560 (3.2)	26 (0.3)	162 (2.5)	211 (13.8)	161 (29.4)	< 0.001
Glucagon-like peptide-1 receptor agonist	67 (0.4)	3 (0.0)	31 (0.5)	22 (1.4)	11 (2.0)	< 0.001
**Comorbidities**						
Hypertension	13,539 (77.3)	6597 (75.6)	5273 (78.7)	1242 (80.2)	427 (77.2)	< 0.001
Diabetes mellitus	7083 (40.4)	1259 (14.4)	3837 (57.3)	1456 (94.1)	531 (96.0)	< 0.001
Dyslipidemia	8246 (47.1)	3502 (40.1)	3520 (52.5)	894 (57.8)	330 (59.7)	< 0.001
Chronic kidney disease	4008 (22.9)	1892 (21.7)	1548 (23.1)	415 (26.8)	153 (27.7)	< 0.001
Cardiac disorders	10,537 (60.1)	5012 (57.4)	4133 (61.7)	1021 (66.0)	371 (67.1)	< 0.001
Myocardial infarction	1182 (6.7)	431 (4.9)	520 (7.8)	165 (10.7)	66 (11.9)	< 0.001
Heart failure	6589 (37.6)	3123 (35.8)	2593 (38.7)	618 (39.9)	255 (46.1)	< 0.001
Cerebrovascular disorders	4185 (23.9)	2012 (23.1)	1618 (24.1)	408 (26.4)	147 (26.6)	0.012
Thrombosis and embolism-related diseases	1691 (9.6)	741 (8.5)	707 (10.6)	180 (11.6)	63 (11.4)	< 0.001
Gastrointestinal disease	5311 (30.3)	2648 (30.3)	2028 (30.3)	473 (30.6)	162 (29.3)	0.955
Active cancer	1913 (10.9)	862 (9.9)	790 (11.8)	193 (12.5)	68 (12.3)	< 0.001
Dementia	1358 (7.7)	700 (8.0)	467 (7.0)	120 (7.8)	71 (12.8)	< 0.001
Fall within 1 year	1270 (7.2)	604 (6.9)	484 (7.2)	124 (8.0)	58 (10.5)	0.006

Data are n (%) or mean ± standard deviation. *P values for comparison among four HbA1c subgroups

*AF* atrial fibrillation, *DOAC* direct oral anticoagulant, *HbA1c* glycated hemoglobin, *TTR* time in the therapeutic range

Patients with HbA1c ≥ 8.0% were significantly more likely to have lower CrCl, higher CHA_2_DS_2_-VASc and HAS-BLED scores, and higher prevalence of non-paroxysmal AF, hypertension, dyslipidemia, chronic kidney disease, cardiovascular disease, thromboembolism-related disease, cerebrovascular disease, malignant tumor, dementia, falls within 1 year, and lower administration of DOACs, but higher administration of warfarin compared with the other subgroups. Regarding oral antidiabetic drug use, patients with HbA1c 7.0% to < 8.0% and those with HbA1c ≥ 8.0% had higher rates for sulfonylureas, alpha-glucosidase inhibitors, DPP-4 inhibitors, SGLT2 inhibitors, and others, as well as higher rates for injectable antidiabetic drugs, including insulin and GLP-1 receptor agonists, compared with the other subgroups (Table 1).

### Study endpoints

**Fig. 1:**
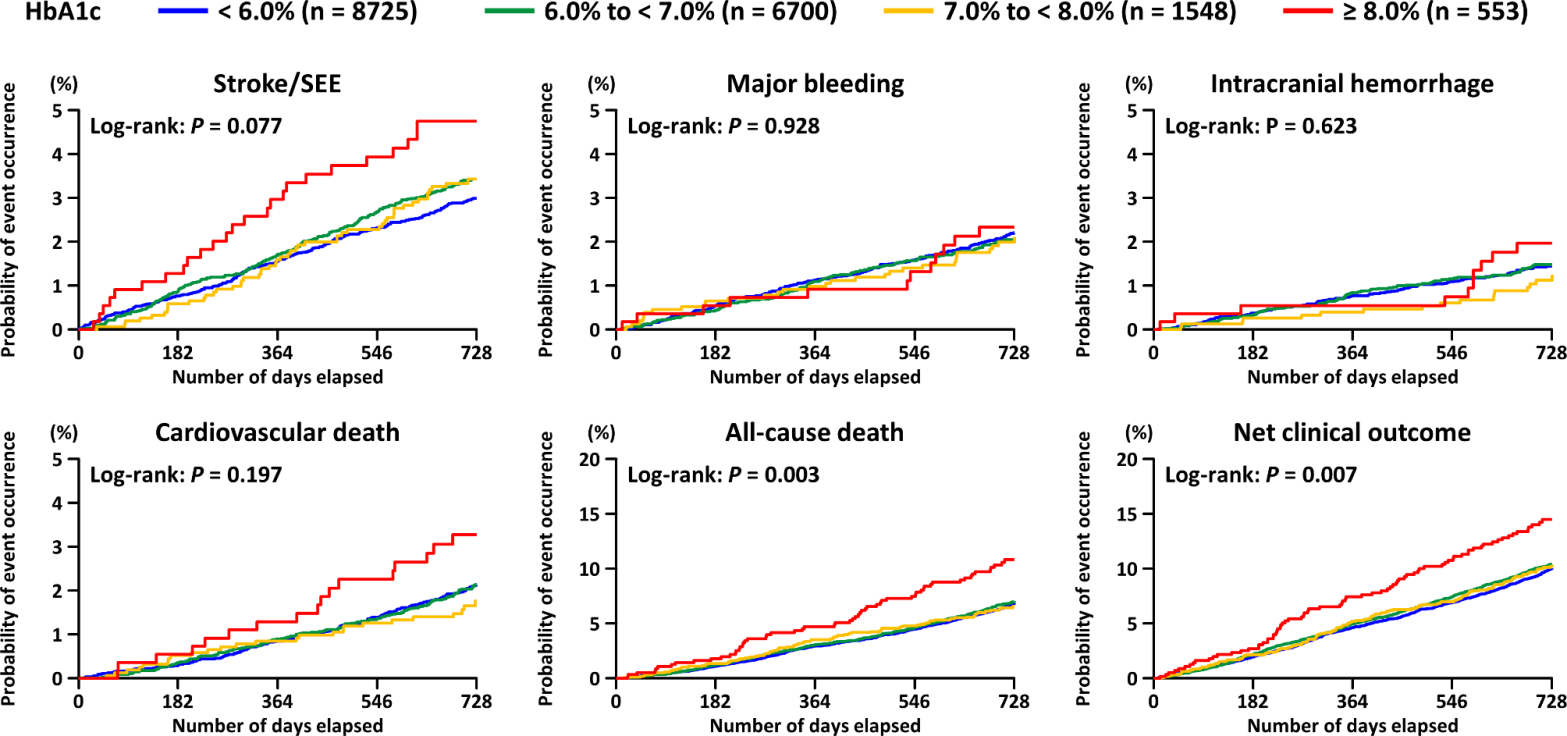
Kaplan–Meier curves for clinical outcomes by HbA1c levels *HbA1c* glycated hemoglobin, *SEE* systemic embolic events

Figure 1 shows the Kaplan–Meier estimates of the probability of occurrences of each event. The probabilities of event occurrences were significantly different among HbA1c categories for all-cause death (log-rank *P* = 0.003) and net clinical outcome (log-rank *P* = 0.007), and both event occurrences were visually higher in patients with HbA1c ≥ 8.0%. A similar trend was observed for stroke/SEE in the HbA1c ≥ 8.0% subgroup, but the difference did not reach statistical significance.

Table 2 summarizes the incidence rates of events by HbA1c level subgroup. Although the incidence of events was comparable among groups with HbA1c < 8.0, a remarkable increase was observed in the HbA1c ≥ 8.0% subgroup, particularly for stroke/SEE (2.50/100 person-years [95% CI: 1.52–3.49]), all-cause death (5.69/100 person-years [95% CI: 4.23–7.16]), and net clinical outcome (7.83/100 person-years [95% CI: 6.09–9.57]).

**Table 2:** Incidence rates of stroke/SEE, major bleeding, ICH, cardiovascular death, all-cause death, and net clinical outcome of 17,526 patients

Event	< 6.0% (n = 8725)		6.0% to < 7.0% (n = 6700)		7.0% to < 8.0% (n = 1548)		≥ 8.0% (n = 553)	
	Number of occurrences (%)	Incidence/100 person-years (95% CI)	Number of occurrences (%)	Incidence/100 person-years (95% CI)	Number of occurrences (%)	Incidence/100 person-years (95% CI)	Number of occurrences (%)	Incidence/100 person-years (95% CI)
Stroke/SEE	247 (2.83)	1.53 (1.34–1.72)	218 (3.25)	1.76 (1.52–1.99)	50 (3.23)	1.74 (1.26–2.23)	25 (4.52)	2.50 (1.52–3.49)
Major bleeding	181 (2.07)	1.11 (0.95–1.28)	130 (1.94)	1.04 (0.86–1.22)	30 (1.94)	1.04 (0.67–1.42)	12 (2.17)	1.18 (0.51–1.85)
ICH	119 (1.36)	0.73 (0.60–0.86)	94 (1.40)	0.75 (0.60–0.90)	17 (1.10)	0.59 (0.31–0.87)	10 (1.81)	0.98 (0.37–1.59)
Cardiovascular death	41 (0.47)	0.25 (0.17–0.33)	23 (0.34)	0.18 (0.11–0.26)	4 (0.26)	0.14 (0.00–0.27)	4 (0.72)	0.39 (0.01–0.78)
All-cause death	568 (6.51)	3.47 (3.18–3.75)	450 (6.72)	3.58 (3.25–3.91)	98 (6.33)	3.38 (2.71–4.05)	58 (10.49)	5.69 (4.23–7.16)
Net clinical outcome†	840 (9.63)	5.21 (4.86–5.56)	675 (10.07)	5.47 (5.06–5.89)	154 (9.95)	5.40 (4.55–6.26)	78 (14.10)	7.83 (6.09–9.57)

^†^Net clinical outcome was a composite of stroke/SEE, major bleeding, and all-cause deaths

*CI* confidence interval, *ICH* intracranial hemorrhage, *SEE* systemic embolic events

**Fig. 2:**
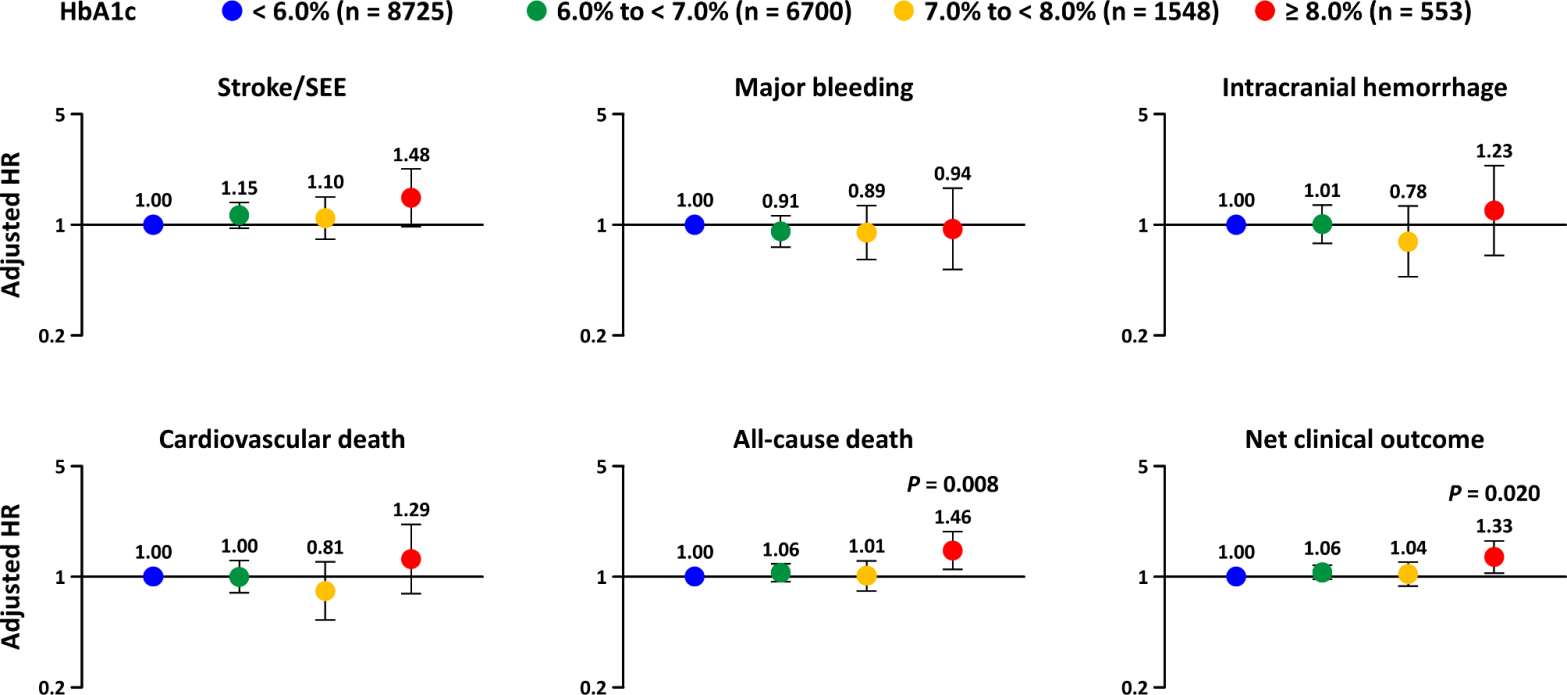
Multivariate analysis using Cox proportional hazards models by HbA1c levels *HbA1c < 6.0% was the reference; bars represent 95% confidence interval* *HbA1c* glycated hemoglobin, *HR* hazard ratio, *SEE* systemic embolic events

Figure 2 shows the results of multivariate analysis using the Cox proportional hazards model by HbA1c, with HbA1c < 6.0% as reference. The risk (aHR [95% CI]) of stroke/SEE (1.48 [0.97–2.25]) was numerically high at HbA1c ≥ 8.0%. The risks of all-cause death (1.46 [1.11–1.93]) and net clinical outcome (1.33 [1.05–1.68]) were significantly higher in the HbA1c ≥ 8.0% subgroup, while there were no significant differences in the risks of major bleeding (0.94 [0.52–1.70]) or other outcomes. No significant difference was observed in the risk of any event, with HbA1c levels ranging between 6.0% to < 7.0% and 7.0% to < 8.0%. Supplementary Table 1 shows the multivariate analysis using a Cox proportional hazards model in patients with AF and diagnosed with diabetes by each participating physician’s judgement by HbA1c, with HbA1c 6.0% to < 7.0% as reference. In patients with HbA1c ≥ 8.0%, significantly higher risk was observed for all-cause death (1.47 [1.10–1.97]).

Table 3 shows the multivariate analysis using the Cox proportional hazards model by HbA1c, no OAC, and OAC treatment. Among patients with HbA1c 6.0% to < 7.0%, DOAC use was associated with significantly lower risk (aHR [95% CI]) of stroke/SEE (0.71 [0.53–0.95]), major bleeding (0.56 [0.39–0.82]), intracranial hemorrhage (0.55 [0.36–0.85]), and net clinical outcome (0.80 [0.68–0.95]) compared with warfarin. For patients with HbA1c ≥ 8.0%, there was a higher risk of cardiovascular death (5.52 [1.03–29.62]) and net clinical outcome (1.69 [0.96–2.97]) with DOAC use. However, no interaction was observed between HbA1c level and anticoagulants. In Supplementary Tables 2, we show the number (%) of events by HbA1c level and anticoagulant treatment (i.e., warfarin, no OAC, or DOAC).

**Table 3:** Multivariate analysis using Cox proportional hazards models with DOACs (n = 11,921) and No OAC (n = 1262) versus warfarin (n = 4336) by HbA1c levels

	Total	HbA1c, %				Interaction *P*-value^††^
		< 6.0 (n = 8721)	6.0 to < 7.0 (n = 6699)	7.0 to < 8.0 (n = 1548)	≥ 8.0 (n = 551)	
	HR^†^ (95% CI)	HR^†^ (95% CI)	HR^†^ (95% CI)	HR^†^ (95% CI)	HR^†^ (95% CI)	
**Stroke/SEE** DOAC	0.84 (0.69–1.02)	0.97 (0.71–1.32)	0.71 (0.53–0.95)	0.77 (0.40–1.45)	1.75 (0.63–4.87)	0.377
No OAC	1.17 (0.83–1.67)	1.29 (0.79–2.11)	1.30 (0.73–2.30)	0.66 (0.14–3.05)	1.10 (0.11–11.26)	
**Major bleeding** DOAC	0.81 (0.64–1.03)	1.05 (0.73–1.50)	0.56 (0.39–0.82)	0.96 (0.40–2.29)	1.04 (0.27–4.00)	0.406
No OAC	0.78 (0.48–1.25)	0.94 (0.49–1.80)	0.67 (0.29–1.51)	0.48 (0.06–4.10)	4.14 (0.27–63.78)	
**ICH** DOAC	0.76 (0.57–1.02)	1.04 (0.66–1.62)	0.55 (0.36–0.85)	0.61 (0.19–1.95)	0.74 (0.17–3.31)	0.671
No OAC	0.60 (0.32–1.13)	0.81 (0.36–1.83)	0.52 (0.18–1.52)	0.00 (-, -)	0.00 (-, -)	
**CV death** DOAC	0.84 (0.67–1.07)	0.79 (0.56–1.11)	0.76 (0.52–1.11)	0.83 (0.33–2.06)	5.52 (1.03–29.62)	0.420
No OAC	1.50 (1.02–2.21)	1.15 (0.67–1.97)	2.02 (1.08–3.78)	0.92 (0.10–8.20)	19.06 (1.56–233.10)	
**All-cause death** DOAC	0.87 (0.76–0.99)	0.79 (0.66–0.96)	0.91 (0.74–1.12)	0.81 (0.52–1.28)	1.49 (0.77–2.90)	0.367
No OAC	1.39 (1.12–1.73)	1.39 (1.04–1.87)	1.42 (0.97–2.06)	0.63 (0.21–1.88)	3.50 (1.14–10.74)	
**Net clinical outcome** DOAC	0.87 (0.74–1.03)	0.86 (0.77–0.96)	0.80 (0.68–0.95)	0.85 (0.59–1.22)	1.69 (0.96–2.97)	0.103
No OAC	1.26 (1.05–1.52)	1.31 (1.01–1.69)	1.32 (0.96–1.81)	0.56 (0.23–1.36)	3.09 (1.13–8.42)	

^†^The adjusted hazard ratio of DOAC or No OAC to warfarin (reference). Adjustment factors were age, sex, body mass index, history of bleeding, type of AF, systolic blood pressure, severe hepatic disease, hyperuricemia, heart failure and/or reduced left ventricular ejection fraction, myocardial infarction, cerebrovascular disease, thromboembolic disease, active cancer, dementia, fall within 1 year, history of catheter ablation, creatinine clearance, digestive diseases, polypharmacy, and use of antiarrhythmic drugs, anti-platelet agents, proton pump inhibitors, P-glycoprotein inhibitors, and anti-hyperlipidemia drugs

^††^*P*-value for the interaction of anticoagulant type with HbA1c.

*AF* atrial fibrillation, *CI* confidence interval, *CV* cardiovascular, *DOAC* direct oral anticoagulant, *HbA1c* glycated hemoglobin, *HR* hazard ratio, *ICH* intracranial hemorrhage, *OAC* oral anticoagulant, *SEE* systemic embolic events

Table 4 shows the multivariate analysis using Cox proportional hazards models by HbA1c and oral and injectable DM medication in patients with DM. Overall, using the subgroup with no oral antidiabetic drug use as a reference, oral antidiabetic drug use was not associated with risk reduction. Similar results were observed for patients receiving injectable antidiabetic drugs. No significant difference was observed in event risk with versus without injectable antidiabetic drugs.

**Table 4:** Multivariate analysis of 8733 diabetes patients using Cox proportional hazards models with oral and injectable antidiabetic medication

	Total^†^	HbA1c, %				Interaction *P*-value^†††^
		< 6.0 (n = 1259)	6.0 to < 7.0 (n = 3837)	7.0 to < 8.0 (n = 1456)	≥ 8.0 (n = 531)	
	HR^†^ (95% CI)	HR^††^ (95% CI)	HR^††^ (95% CI)	HR^††^ (95% CI)	HR^††^ (95% CI)	
**With oral antidiabetic drugs (n = 4740), reference: no oral antidiabetic drug use (n = 3993)**						
Stroke/SEE	0.99 (0.77–1.28)	1.18 (0.60–2.31)	0.84 (0.59–1.19)	1.83 (0.78–4.29)	1.36 (0.43–4.27)	0.547
Major bleeding	1.14 (0.84–1.56)	1.54 (0.61–3.93)	1.01 (0.64–1.60)	0.71 (0.30–1.68)	4.41 (0.49–40.17)	0.470
ICH	1.06 (0.73–1.55)	1.90 (0.55–6.50)	0.72 (0.42–1.21)	0.71 (0.21–2.41)	NC^††††^	0.343
CV death	0.96 (0.70–1.32)	1.72 (0.69–4.28)	0.68 (0.44–1.06)	1.84 (0.58–5.84)	4.88 (0.42–56.31)	0.120
All-cause death	0.91 (0.77–1.08)	0.95 (0.61–1.48)	0.78 (0.61–1.00)	0.76 (0.47–1.23)	1.46 (0.69–3.09)	0.078
Net clinical outcome	0.96 (0.83–1.10)	1.07 (0.74–1.54)	0.82 (0.67–1.00)	0.88 (0.59–1.30)	1.50 (0.79–2.84)	0.108
**With injectable antidiabetic drugs (n = 678), reference: no injectable antidiabetic drug use (n = 8055)**						
Stroke/SEE	0.80 (0.50–1.28)	1.16 (0.15–9.04)	0.48 (0.15–1.52)	1.09 (0.47–2.51)	0.64 (0.24–1.72)	0.840
Major bleeding	0.86 (0.48–1.55)	NC^††††^	1.36 (0.54–3.42)	0.95 (0.31–2.90)	0.06 (0.00–0.72)	0.546
ICH	0.67 (0.30–1.46)	NC^††††^	1.11 (0.34–3.61)	NC^††††^	0.05 (0.00–1.44)	0.596
CV death	0.58 (0.31–1.10)	NC^††††^	0.44 (0.11–1.80)	0.71 (0.20–2.58)	0.55 (0.11–2.72)	0.973
All-cause death	1.02 (0.77–1.36)	0.74 (0.18–3.08)	1.31 (0.79–2.15)	1.31 (0.77–2.22)	1.04 (0.56–1.93)	0.419
Net clinical outcome	1.03 (0.81–1.30)	0.71 (0.22–2.26)	1.20 (0.78–1.84)	1.31 (0.86–1.99)	0.87 (0.51–1.47)	0.449

^†^Adjusted by the same factors as in Table 3 plus subgroups of HbA1c.

^††^Adjusted by the same factors as in Table 3

^†††^The *P*-value for the interaction of oral diabetes medication and HbA1c levels

^††††^NC: The number of events was too small to calculate

*AF* atrial fibrillation, *CI* confidence interval, *CV* cardiovascular, *DOAC* direct oral anticoagulant, *HbA1c* glycated hemoglobin, *HR* hazard ratio, *ICH* intracranial hemorrhage, *NC* not calculated, *SEE* systemic embolic events

## Discussion

This is the first study to evaluate the incidence of the primary outcomes by HbA1c level and the efficacy and safety of OAC in elderly NVAF patients aged ≥ 75 years. The main findings of this sub-analysis were as follows. First, patients in the HbA1c ≥ 8.0% subgroup had significantly higher probabilities of event occurrence for all-cause death and net clinical outcome. A similar trend was observed for stroke/SEE, but the differences did not reach statistical significance. Multivariate analysis of HbA1c (< 6.0% as reference) did not show significant differences in the risk of any event with HbA1c levels ranging between 6.0% to < 7.0% and those between 7.0% and < 8.0%; however, HbA1c ≥ 8.0% was significantly associated with increased risk of all-cause death and net clinical outcome. Although recent data from Japan [19, 20] indicate that diabetes was not a risk factor for stroke in AF patients, our results suggest that event risk is indeed influenced by blood glucose levels. Second, multivariate analysis using the Cox proportional hazards model showed a lower event risk of cardiovascular death and net clinical outcome for DOAC use (compared with warfarin) among patients in the HbA1c 6.0% to < 7.0% subcategory but a higher risk of these events among patients with HbA1c ≥ 8.0%. Nevertheless, there was no significant interaction between HbA1c and OAC medication. The lack of significant interaction may be attributable to the extremely low number of events among patients with HbA1c ≥ 8.0%, and there is a high possibility that the observed data were due to chance. Further, this result is comparable with that shown in the analysis of each event by OAC treatment for the overall population, in which the risks of stroke/SEE, all-cause death, and net clinical outcomes were lower in the DOAC group vs. warfarin [17]. Third, regarding the risk of each event with oral or injectable antidiabetic drug use, neither oral nor injectable antidiabetic drugs were associated with risk reduction. Finally, the present findings show that the HbA1c threshold, which is the target of diabetes treatment, is appropriate even for elderly patients with NVAF, which has important implications for clinical practice.

Previous studies have reported that the risk of ischemic stroke in AF patients with DM is more closely related to the disease duration of DM than to glycemic control based on HbA1c [21]. In contrast, in this sub-analysis, HbA1c ≥ 8.0% compared with HbA1c < 6.0% had a numerically increased risk of stroke/SEE, which aligns with findings in a previous study [22]. Of note, we did not assess the effect of disease duration on evaluated clinical outcomes. The present results are also consistent with a previous report in which the incidence of all-cause death increased with high HbA1c levels [23]. According to the current Japanese Clinical Practice Guidelines for Diabetes, the HbA1c target for glycemic control among elderly patients with DM aged ≥ 65 years is < 8.0% if intensification of treatment is difficult [10]. The results of this sub-analysis align with the recommended HbA1c target and suggest that this cutoff is also applicable for elderly patients with DM who have concomitant NVAF. Furthermore, many patients with HbA1c ≥ 8.0% were being treated with warfarin rather than DOACs. The reason why these patients were treated preferentially with warfarin may be attributable to the fact that many patients had decreased renal function. Per current treatment guidelines, vitamin K antagonists remain the first-line treatment for preventing thromboembolic events in patients with chronic kidney disease who require anticoagulation [24].

The main limitations of the ANAFIE Registry are primarily associated with the observational design and have been previously reported [16, 17]. Although the overall ANAFIE Registry population comprised over 32,000 elderly patients with NVAF, approximately 15,000 patients did not have data on HbA1c measurements at baseline; however, no significant difference was observed in patient characteristics and probability of event occurrence between those with and those without HbA1c measurement. As this sub-analysis evaluated HbA1c at baseline, we could not assess changes in HbA1c levels over time. Patients with high HbA1c at baseline were included in this sub-analysis, but in some cases, HbA1c may have been measured even though the patient had not been clinically diagnosed with DM. The disease duration of DM was not considered in the present analysis. Rates of use of DPP-4 and SGLT2 inhibitors were low in Japan when the study was conducted. However, these antidiabetic drugs are now widely used, which limits extrapolation of these results to current clinical practice. Finally, HbA1c ≥ 8.0% was evaluated in 553 cases, and this limited number of cases, along with the duration of the observation period, may have precluded an accurate evaluation of the incidence of events.

## Conclusions

Among elderly Japanese patients with NVAF, HbA1c ≥ 8.0% was associated with an increased risk of all-cause death, but the risk of the events did not increase with HbA1c levels of 6.0% to < 8.0% as compared with < 6.0%. Furthermore, there was no significant interaction between HbA1c and OAC medication. Neither oral nor injectable antidiabetic drugs were associated with clinical outcome risk reduction. Individualized treatment goals and strategies based on comorbidities and existing treatments—considering polypharmacy and drug–drug interactions—along with close monitoring and strict control of DM, are crucial for preventing such outcomes and for improving prognosis in this population.

## Electronic supplementary material

Below is the link to the electronic supplementary material.

Supplementary Material 1

## Data Availability

1. Will the individual deidentified participant data (including data dictionaries) be shared? →Yes. 2. What data in particular will be shared? →Individual participant data that underlie the results reported in this article, after deidentification (text, tables, figures, and appendices). 3. Will any additional, related documents be available? If so, what is it? (e.g., study protocol, statistical analysis plan, etc.) →Study Protocol. 4. When will the data become available and for how long? →Ending 36 months following article publication. 5. By what access criteria will the data be shared (including with whom)? →The access criteria for data sharing (including requests) will be decided by a committee led by Daiichi-Sankyo. 6. For what types of analyses, and by what mechanism will the data be available? →Any purpose: Proposals should be directed to yamt-tky@umin.ac.jp. To gain access, data requestors will need to sign a data access agreement.

## List of Abbreviations

AF: Atrial fibrillation
aHR: Adjusted hazard ratio
ANAFIE: All Nippon AF in the Elderly
CI: Confidence interval
CrCl: Creatinine clearance
DM: Diabetes mellitus
DOAC: Direct oral anticoagulant
DPP-4: Dipeptidyl peptidase-4
GLP-1: Glucagon-like peptide-1
HbA1c: Glycated hemoglobin
HR: Hazard ratio
NVAF: Non-valvular atrial fibrillation
OAC: Oral anticoagulant
SEE: Systemic embolic events
SGLT2: Sodium glucose co-transporter 2
