# Supplementary material for: Impact of glycated hemoglobin on 2-year clinical outcomes in elderly patients with atrial fibrillation: sub-analysis of ANAFIE Registry, a large observational study
Source: Cardiovasc Diabetol. 2023 Jul 12;22:175. doi: 10.1186/s12933-023-01915-3 (PMC10339633; doi:10.1186/s12933-023-01915-3)
Supplement: Supplementary file 1 — Supplementary Material 1 [file 12933_2023_1915_MOESM1_ESM.docx]

**Supplementary Table 1.** Multivariate analysis by Cox proportional hazards model among patients with diabetes by HbA1c

| **Event** | **Item** | | **N** | **Event (%)** | | **Multivariate #** | | |
| --- | --- | --- | --- | --- | --- | --- | --- | --- |
|  |  |  |  |  |  | **HR (95% CI)** | | ***P-*value** |
| **Stroke/systemic embolism** | HbA1c | |  |  |  |  |  |  |
|  |  | < 6.0% | 1259 | 45 | (3.6) | 0.93 | (0.66, 1.31) | 0.668 |
|  |  | 6.0% to < 7.0%* | 3837 | 136 | (3.5) |  | - |  |
|  |  | 7.0% to < 8.0% | 1456 | 45 | (3.1) | 0.87 | (0.62, 1.23) | 0.438 |
|  |  | ≥ 8.0% | 531 | 24 | (4.5) | 1.21 | (0.78, 1.88) | 0.389 |
| **Major bleeding** | HbA1c | |  |  |  |  |  |  |
|  |  | < 6.0% | 1259 | 26 | (2.1) | 0.90 | (0.57, 1.42) | 0.665 |
|  |  | 6.0% to < 7.0%* | 3837 | 79 | (2.1) |  | - |  |
|  |  | 7.0% to < 8.0% | 1456 | 28 | (1.9) | 0.98 | (0.63, 1.51) | 0.915 |
|  |  | ≥ 8.0% | 531 | 12 | (2.3) | 1.04 | (0.56, 1.91) | 0.911 |
| **ICH** | HbA1c | |  |  |  |  |  |  |
|  |  | < 6.0% | 1259 | 16 | (1.3) | 0.75 | (0.43, 1.32) | 0.322 |
|  |  | 6.0% to < 7.0%* | 3837 | 60 | (1. 6) |  | - |  |
|  |  | 7.0% to < 8.0% | 1456 | 16 | (1.1) | 0.74 | (0.43, 1.30) | 0.297 |
|  |  | ≥ 8.0% | 531 | 10 | (1.9) | 1.25 | (0.63, 2.45) | 0.523 |
| **CV death** | HbA1c | |  |  |  |  |  |  |
|  |  | < 6.0% | 1259 | 26 | (2.1) | 0.78 | (0.50, 1.22) | 0.277 |
|  |  | 6.0% to < 7.0%* | 3837 | 84 | (2.2) |  | - |  |
|  |  | 7.0% to < 8.0% | 1456 | 24 | (1.6) | 0.77 | (0.49, 1.22) | 0.261 |
|  |  | ≥ 8.0% | 531 | 17 | (3.2) | 1.27 | (0.75, 2.16) | 0.380 |
| **All-cause death** | HbA1c | |  |  |  |  |  |  |
|  |  | < 6.0% | 1259 | 102 | (8.1) | 1.06 | (0.84, 1.34) | 0.642 |
|  |  | 6.0% to < 7.0%* | 3837 | 260 | (6.8) |  | - |  |
|  |  | 7.0% to < 8.0% | 1456 | 90 | (6.2) | 0.93 | (0.73, 1.19) | 0.579 |
|  |  | ≥ 8.0% | 531 | 58 | (10.9) | 1.47 | (1.10, 1.97) | 0.009 |
| **Net clinical outcome** | HbA1c | |  |  |  |  |  |  |
|  |  | < 6.0% | 1259 | 154 | (12.2) | 1.07 | (0.88, 1.29) | 0.513 |
|  |  | 6.0% to < 7.0%* | 3837 | 397 | (10.3) |  | - |  |
|  |  | 7.0% to < 8.0% | 1456 | 142 | (9.8) | 0.95 | (0.78, 1.15) | 0.592 |
|  |  | ≥ 8.0% | 531 | 77 | (14.5) | 1.27 | (0.99, 1.63) | 0.057 |

*Reference

^#^ Adjusted by the same factors as in Table 3 plus anticoagulants.

*CI* confidence interval, *CV* cardiovascular, *HbA1c* glycated hemoglobin, *HR* hazard ratio, *ICH* intracranial hemorrhage.

**Supplementary Table 2**. Number (%) of events by HbA1c level in patients receiving warfarin (n = 4336), no OAC (n = 1262), and DOACs (n = 11,921)

|  | **Total** | **HbA1c, %** | | | |
| --- | --- | --- | --- | --- | --- |
|  |  | **< 6.0** | **6.0 to < 7.0** | **7.0 to < 8.0** | **≥ 8.0** |
|  | N  Event (%) | N  Event (%) | N  Event (%) | N  Event (%) | N  Event (%) |
| **Stroke/ SEE**  Warfarin | 4336  160 (3.69) | 1918  58 (3.02) | 1836  80 (4.36) | 410  16 (3.90) | 172  6 (3.49) |
| No OAC | 1262  44 (3.49) | 725  25 (3.45) | 413  16 (3.87) | 90  2 (2.22) | 34  1 (2.94) |
| DOAC | 11,921  336 (2.82) | 6078  164 (2.70) | 4450  122 (2.74) | 1048  32 (3.05) | 345  18 (5.22) |
| **Major bleeding**  Warfarin | 4336  107 (2.47) | 1918  42 (2.19) | 1836  53 (2.89) | 410  8 (1.95) | 172  4 (2.33) |
| DOAC | 1262  22 (1.74) | 725  13 (1.79) | 413  7 (1.69) | 90  1 (1.11) | 34  1 (2.94) |
| No OAC | 11,921  224 (1.88) | 6078  126 (2.07) | 4450  70 (1.57) | 1048  21 (2.00) | 345  7 (2.03) |
| **ICH**  Warfarin | 4336  75 (1.73) | 1918  27 (1.41) | 1836  39 (2.12) | 410  5 (1.22) | 172  4 (2.33) |
| No OAC | 1262  12 (0.95) | 725  8 (1.10) | 413  4 (0.97) | 90  0 (0.00) | 34  0 (0.00) |
| DOAC | 11,921  153 (1.28) | 6078  84 (1.38) | 4450  51 (1.15) | 1048  12 (1.15) | 345  6 (1.74) |
| **CV death**  Warfarin | 4336  117 (2.70) | 1918  55 (2.87) | 1836  50 (2.72) | 410  9 (2.20) | 172  3 (1.74) |
| No OAC | 1262  38 (3.01) | 725  20 (2.76) | 413  15 (3.63) | 90  1 (1.11) | 34  2 (5.88) |
| DOAC | 11,921  194 (1.63) | 6078  99 (1.63) | 4450  68 (1.53) | 1048  15 (1.43) | 345  12 (3.48) |
| **All-cause death**  Warfarin | 4336  368 (8.49) | 1918  169 (8.81) | 1836  151 (8.22) | 410  33 (8.05) | 172  15 (8.72) |
| No OAC | 1262  118 (9.35) | 725  69 (9.52) | 413  39 (9.44) | 90  4 (4.44) | 34  6 (17.65) |
| DOAC | 11,921  687 (5.76) | 6078  330 (5.43) | 4450  260 (5.84) | 1048  61 (5.82) | 345  36 (10.43) |
| **Net clinical outcome**  Warfarin | 4336  533 (12.29) | 1918  228 (11.89) | 1836  237 (12.91) | 410  49 (11.95) | 172  19 (11.05) |
| No OAC | 1262  156 (12.36) | 725  89 (12.28) | 413  54 (13.08) | 90  6 (6.67) | 34  7 (20.59) |
| DOAC | 11,921  1057 (8.87) | 6078  523 (8.60) | 4450  384 (8.63) | 1048  99 (9.45) | 345  51 (14.78) |

*CV* cardiovascular, *DOAC* direct oral anticoagulant, *HbA1c* glycated hemoglobin, *ICH* intracranial hemorrhage, *OAC* oral anticoagulant*, SEE* systemic embolic events.
